# An Ex Vivo 3D Co-Culture Model of the Bone Marrow Niche Better Predicts Clinically Relevant Epigenetic Target Modulation to Improve Immunotherapy in Multiple Myeloma

**DOI:** 10.3390/cancers18193093

**Published:** 2026-09-23

**Authors:** Christine Greil, Johannes Jung, Julia Felthaus, Dagmar Wider, Monika Engelhardt, Ralph Wäsch

**Affiliations:** 1 Department of Hematology, Oncology and Stem Cell Transplantation, Medical Center—University of Freiburg, Faculty of Medicine, University of Freiburg, 79106 Freiburg, Germany; 2 Clinic for Hematology and Medical Oncology, University Medical Center Goettingen, 37075 Goettingen, Germany

**Keywords:** immunotherapy, multiple myeloma, CD38 expression, epigenetic modulation, 3D model, stromal cell support

## Abstract

**Background**: In the last decade, treatment options for multiple myeloma have increased remarkably, including antibodies or CAR-T cells targeting myeloma surface markers with unprecedented efficacy. To enhance immunomodulation and clinical efficacy even more, the combination of these agents with other drugs is highly relevant. A significant association between CD38 expression on myeloma cells and the cytotoxicity of the anti-CD38 antibody daratumumab has been shown. All-*trans* retinoic acid, the pan-histone deacetylase inhibitor panobinostat, and the enhancer of the zeste homolog 2 inhibitor tazemetostat induce CD38 upregulation and can augment the anti-myeloma effect of daratumumab in conventional cell culture. However, translation into a clinical trial failed. **Methods**: To better predict in vivo drug effects preclinically, we used an ex vivo 3D model, allowing long-term propagation of myeloma cell lines and primary cells from 12 patients in co-culture with human stromal cells in a milieu that more closely resembles the physiological conditions in the bone marrow niche. **Results**: While epigenetic CD38 upregulation with different drugs leading to enhanced daratumumab efficacy was reproducible in conventional 2D culture, this effect was not confirmed in the optimized 3D model. Here, the epigenetic modification of CD38 expression and the impact on the cytotoxic effect of daratumumab seem to depend on the individual plasma cell and its interaction with the bone marrow microenvironment, which could be a plausible explanation for the lack of clinical efficacy of this combination treatment. **Conclusions**: Epigenetically induced CD38 upregulation to enhance the effect of anti-CD38 antibodies does not appear to be clinically relevant when using an optimized preclinical model.

## Simple Summary

A 3D co-culture system for myeloma cells with stromal cell support is a more realistic model of the bone marrow niche and can better predict the effects of various anti-myeloma substances and their expected combinations in vivo. It was shown in conventional 2D cell culture that epigenetic modifiers such as ATRA increase CD38 expression on plasma cells and enhance the cytotoxic effect of anti-CD38 antibodies such as daratumumab in multiple myeloma. This could not be confirmed in the 3D co-culture system. Here, the epigenetic modification of CD38 expression and the impact on the cytotoxic effect of daratumumab in myeloma seem to depend on the individual plasma cell and its interaction with the bone marrow microenvironment, which could be a plausible explanation for the recently shown lack of clinical efficacy of this combination treatment.

## 1. Introduction

Multiple myeloma (MM) is a cytogenetically heterogeneous plasma cell dyscrasia characterized by the clonal proliferation of malignant plasma cells in the bone marrow (BM) producing monoclonal immunoglobulin. In the last decade, treatment options for MM have increased remarkably. Despite the introduction of targeted immunotherapies, including monoclonal antibodies, antibody–drug conjugates, bispecific antibodies, and chimeric antigen receptor (CAR) T cells, MM is still associated with a substantial burden of morbidity and mortality [1,2,3,4], requiring novel therapeutic approaches. To enhance the immunomodulation and clinical efficacy of single substances, the combination of different agents seems highly relevant.

CD38 is a type II transmembrane glycoprotein that is particularly and highly expressed in MM and on healthy plasma cells [5]. This specific expression profile allows it to more selectively target MM cells with the use of monoclonal antibodies as the first targeted immunotherapy. The first-in-class agent here was the anti-CD38 monoclonal antibody daratumumab.

Daratumumab was initially applied to the treatment of relapsed and refractory MM [6,7], and is now a standard component of first-line therapy [8], as well as for maintenance [9] and in smoldering MM [10]. It is usually applied as combination therapy, as the remission rate under monotherapy is lower, and the duration of response is shorter [11].

Daratumumab provides multiple mechanisms of action, including complement-dependent cytotoxicity (CDC) and natural killer (NK) cell-mediated antibody-dependent cellular cytotoxicity (ADCC) through Fc-receptor-dependent mechanisms of effector cells [12]. Additionally, it alters the immunological landscape through an increase in T-helper and cytotoxic T cells in the peripheral blood (PB) and BM of MM patients.

It has been demonstrated that pretreatment CD38 expression on MM cells was lower in patients with insufficient therapy response and further declined after daratumumab treatment [13]. Therefore, modulating the function of proteins involved in the regulation of CD38 expression on plasma cells seems to be a promising approach to further enhance the efficiency of daratumumab. Several substances have been tested preclinically to increase CD38 expression. The epigenetic modulator all-*trans* retinoic acid (ATRA) has been shown to increase CD38 levels on MM cells and enhance daratumumab-mediated CDC and ADCC [13,14,15,16].

The pan-histone deacetylase inhibitor (HDACi) panobinostat has intrinsic anti-myeloma activity [17]. It modulates gene expression in lymphoid and myeloid malignancies via epigenetic changes and leads to enhanced expression of CD20, CD33, and CD38 [18,19]. The small-molecule enhancer of zeste homolog 2 (EZH2) inhibitor tazemetostat, a methyltransferase inhibitor and antineoplastic agent used in the therapy of advanced epithelioid sarcoma and follicular lymphoma, also induces CD38 upregulation [20,21]. An increase in CD38 expression has also been reported after lenalidomide and ruxolitinib treatment [22,23]. Thus, upregulation of CD38 expression through such combinations should enhance the clinical effect of daratumumab. It has been shown that both ATRA and panobinostat induce CD38 upregulation and augment the anti-myeloma effect of daratumumab in conventional 2D monocultures of cell lines and primary MM samples [16,19]. However, in a phase I/II study with pretreated MM patients, ATRA induced a transient increase in CD38 expression in PB immune cell subsets, but the trial did not achieve its primary clinical endpoint, with a dismal overall response rate (ORR) for the combination of daratumumab and ATRA of only 5% [24].

These observations suggest that more realistic in vitro models are needed that can better predict the clinical effect of preclinically active anti-myeloma substances. However, most of the available MM mouse models do not reflect the heterogeneity of the human disease, as they are based on certain mutations that individually promote the expansion and malignant transformation of plasma cells but do not consider the molecular characteristics of the various MM subtypes [25]. Moreover, the transplantation of human myeloma poses the challenge of low engraftment, as well as the artificial interaction between the mouse microenvironment and human cells [26].

MM cells depend on their interaction with the microenvironment of the BM [27], and primary MM cells do not expand in ex vivo monocultures outside of this functional BM niche [28], although they require cytokine support [29]. Thus, in vitro models should simulate the biological mechanisms mediated by stromal cells in influencing tumor biology and immunomodulation. Survival and growth of primary human MM cells have been demonstrated in co-culture with primary BM mesenchymal cells [30] and immortalized stromal cell lines, such as HS-5 and HS-27 [31]. Our group established a conical agarose ex vivo 3D co-culture platform to allow long-term propagation of MM cells, especially primary patient cells, in distance co-culture with BM stromal cells over a significantly longer period than in conventional culture [29]. This system allows us to examine the cytotoxic effects of different substances in a more realistic in vitro model with a milieu more resembling the physiological conditions in the BM niche to explore the effect of single agents and their combinations and to better predict the expected clinical efficacy of preclinically active substances [29].

## 2. Materials and Methods

### 2.1. Cell Lines, Cell Culture, and Agents

The MM cell lines MM-1S (ATCC through the official distribution partner, LGC Standards, Wesel, Germany), OPM-2, and L363 (both from DSMZ, Braunschweig, Germany), with varying levels of basal CD38 expression and the human stromal cell lines HS-5 and HS-27 (former Oncotest, now Charles River Laboratories, Sulzfeld, Germany), were cultured at 37 °C in a humidified incubator containing 5% CO_2_, with RPMI 1640 medium (Life Technologies GmbH, Darmstadt, Germany), 10–15% fetal calf serum (FCS; Sigma-Aldrich Chemie GmbH, Taufkirchen, Germany), 1% penicillin/streptomycin, and 1% sodium pyruvate (Life Technologies GmbH, Darmstadt, Germany). NK92-16A-GFP cells (provided by B. Walchek [32]) were cultured in MEM alpha medium (Life Technologies GmbH, Darmstadt, Germany) with 15% FCS, 1% sodium pyruvate, 55 µM beta-mercaptoethanol, and 200 units of IL-2 recombinant protein (Pepro Tech, Cranbury, NJ, USA). All cell lines tested negative for mycoplasma contamination.

For 2D co-culture, stromal and myeloma cells were cultured in a shared dish in a 1:1 ratio. For the supernatant experiments, HS-5 was cultured as described above. The medium was removed after three days of culture, filtered through a syringe filter (25 mm, 0.45 µm), and diluted with the medium as indicated before being added to the myeloma cell culture.

For 3D culture, CoSeedis™ (abc biopply AG, Solothurn, Switzerland) was used. It is an agarose-based microwell device manufactured by computer-aided design with conical microwells. MM cells were seeded into and harvested from the cavities using a centrifugation step, as described [29,33]. For distance co-culture, the microwell device, filled with MM cells, was placed on confluent stromal cells.

In the combination experiments, NK92-16A-GFP and the respective MM cells were harvested from their monocultures, thoroughly mixed, and seeded in a 1:1 ratio jointly into the cavities.

Unless other concentrations are indicated, cells were treated with 50 nM ATRA (Sigma-Aldrich Chemie GmbH, Taufkirchen, Germany, catalog number T1850000), 10 nM panobinostat (Selleck Biotechnology GmbH, Munich, Germany, catalog number S1030), 2.4 µM tazemetostat (EPZ-6438; MedChemExpress, Monmouth Junction, NJ, USA, catalog number Hy-13803), and 1 µg/mL daratumumab (Selleck Biotechnology GmbH, Munich, Germany, catalog number A2027) [34,35]. In the monotherapy experiments, ATRA, Panobinostat, and tazemetostat were administered for 48 h. In the combination experiments, cells were pretreated with ATRA at the indicated concentration for 72 h. After removal of the medium, the combination of ATRA and daratumumab was added for another 12 h.

### 2.2. Primary MM Cells

BM samples from 11 patients and the pleural effusion of 1 patient with untreated or relapsed/refractory MM were examined. Mononuclear cells were isolated using density gradient centrifugation (Ficoll), frozen, and stored in liquid nitrogen until further use. A density of 1 × 10^6^ cells per ml was used in all experiments.

For all samples, CD38 positivity was confirmed by immunohistochemical examination of the BM aspirate, as well as a trephine biopsy during clinical routines.

### 2.3. Flow Cytometry

Adherent cells from the 2D culture were detached using trypsin-EDTA (Life Technologies GmbH, Darmstadt, Germany) and washed in 3 mL PBS. Cell pellets from the 3D culture were washed out of the cavities with PBS. Both were transferred to filter tubes and centrifuged at 1200 rpm for 8 min.

Pellets were dissolved and stained in 100 µL staining solution with anti-CD38/FITC (diluted 1:100; Biolegend, San Diego, CA, USA, catalog number 356610); multi-epitope anti-CD38/FITC (diluted 1:20; BD Biosciences, Heidelberg, Germany, catalog number Cyt-38F2) or anti-CD138/PE (diluted 1:40; Becton Dickinson, Heidelberg, Germany, catalog number 550805); anti-mouse IgG1/FITC (diluted 1:100; Biolegend, San Diego, CA, USA, catalog number 400110) or anti-mouse IgG1/PE (diluted 1:40; Becton Dickinson, Heidelberg, Germany, catalog number 555749); and DAPI (0.2 µg/mL; Biotium, Fremont, CA, USA).

Then, 2 × 10^4^ cells were measured on a FACS device (Fortessa, Becton Dickinson, Heidelberg, Germany). Analysis was performed using FlowJo (version 10.3).

For measurement of the mean fluorescence intensity (MFI) of CD38 on MM cells after daratumumab treatment in co-culture with GFP-labeled NK cells, cells were first gated for GFP. Primary MM cells were first gated for CD138 expression.

### 2.4. Statistics and Reproducibility

N-numbers in the figure legends indicate how many independent biological experiments, carried out as technical duplicates, were conducted in the respective setup. To compare the central tendency of two different experimental sets based on samples of unrelated data, the unequal variance *t*-test (Welch’s test) was applied. Data are presented as means and standard deviations. A *p*-value < 0.05 was reported as statistically significant.

## 3. Results

### 3.1. CD38 Expression, Cell Viability, and Response to the Epigenetic Modifier ATRA Vary Depending on the Cell Line and Culture Conditions

Two-dimensional and three-dimensional cultures with and without stromal cell support with HS-5 (Figure 1) were compared in the MM cell lines MM-1S and OPM-2 with comparably high basal CD38 expression [20]. ATRA was used because of its validated impact on CD38 expression under conventional culture conditions [16].

As previously described [16], an increase in CD38 expression could be observed in MM-1S after ATRA treatment independent of cell culture conditions (Figure 2A), although the difference did not reach statistical significance under all culture conditions. Interestingly, as compared to the control, CD38 expression was already 1.5-fold higher after stromal cell support alone. There was no impact of ATRA treatment or cell culture conditions on cell viability (Figure 2B).

In OPM-2, ATRA led to an even stronger increase in CD38 expression under all culture conditions compared to MM-1S (4.7-fold versus 2.2-fold compared to the untreated control in conventional culture). The effect was higher in 2D cell culture and with stromal support (Figure 2C). There was again no impact of ATRA treatment or cell culture conditions on cell viability (Figure 2D).

### 3.2. A Similar Influence of Culture Conditions on CD38 Expression, Cell Viability, and Treatment Response Is Observed After Treatment with the HDACi Panobinostat

In the next step, MM-1S and OPM-2 cells were treated with panobinostat under the culture conditions outlined above (Figure 1).

In MM-1S, the previously described CD38 upregulation after treatment with panobinostat [19] was confirmed in 2D monoculture, albeit it was not significant. This did not occur under co-culture conditions, where higher pre-existing CD38 levels were again achieved using stromal support alone (1.4-fold; Figure 3A). Likewise, in the 3D model, panobinostat did not have a relevant impact on CD38 expression (Figure 3A). The known effect of panobinostat on cell viability was observed independently of this effect on CD38 expression under all culture conditions, but less so in the 3D culture (reduction to 80% of the untreated control in 3D culture versus 50% in 2D culture; Figure 3B).

In OPM-2, panobinostat did not have a relevant effect on CD38 expression, neither under conventional culture conditions nor in the 3D model (Figure 3C). Again, viability was reduced after treatment with panobinostat, independent of culture conditions (Figure 3D).

CD38 upregulation also remained cell-line-dependent after long-term cultivation (Figure S1). In addition, the impact of stromal support on CD38 upregulation was largely independent of the stromal cell line or the concentration of supernatant obtained from stromal cell culture (Figure S2).

### 3.3. ATRA Induces CD38 Upregulation and Enhances the Cytotoxicity of Daratumumab in Conventional 2D Culture, but Has No Significant Impact on Cell Viability in the 3D Model

Since we observed a more pronounced increase in CD38 expression in OPM-2 cells and with ATRA treatment compared to MM-1S and panobinostat, respectively, we selected this cell line and this substance for subsequent combination experiments with the anti-CD38 antibody daratumumab. To account for the possible influence of the cell lines’ basal CD38 expression on upregulation when investigating the effect of daratumumab, as also suggested by Chemlal et al. [20], we additionally examined L363 cells, which exhibit significantly lower basal CD38 expression than OPM-2 and MM-1S cells [20].

Prior treatment with anti-CD38 agents could prevent detection of CD38 expression on the plasma cell surface by flow cytometry, as common anti-CD38 antibodies bind to epitopes overlapping with the daratumumab binding site. As expected, measurements with the multi-epitope antibody revealed higher CD38 values compared to staining with the conventional monoclonal antibody (Figure S3).

Independent of the cell line and the anti-CD38 antibody used for detection, CD38 expression was generally slightly reduced by daratumumab treatment (Figure 4A,C,E,G; Figure S3). As previously described [16], CD38 expression was higher and cell viability lower after combination of daratumumab with ATRA in conventional 2D culture in both OPM-2 and L363 (Figure 4B,D), supporting the propagated influence of CD38 upregulation on the anti-myeloma effect of daratumumab under conventional culture conditions.

In 3D co-culture, CD38 expression increased significantly in daratumumab-treated plasma cells, with high basal expression after the addition of ATRA (OPM-2; Figure 4E), but only slightly in low-expressing cells (L363; Figure 4G). In OPM-2, neither daratumumab alone nor in combination with ATRA had an impact on cell viability (Figure 4F). In L363, daratumumab alone already showed substantial cytotoxicity (reduction of viable cells to 33% of the untreated control in 3D culture, Figure 4H; reduction to 86% in 2D culture, Figure 4D). Interestingly, the combination with ATRA had no significant additional effects (Figure 4H).

EZH2 inhibition with tazemetostat has no relevant influence on CD38 expression or cell viability when combined with daratumumab in 3D co-culture (Figure S4).

### 3.4. In Long-Term 3D Co-Culture, Primary MM Cells Derived from Patient Samples Differ Significantly in Their CD38 Expression, Both Untreated and After the Addition of ATRA or Tazemetostat

Primary plasma cells derived from the BM of MM patients (n = 11) or obtained from pleural effusion (n = 1) were cultured in the 3D matrix with stromal cell support for 9 days and treated with ATRA.

Clinical parameters are depicted in Table 1. The median age was 64 years, and 70% of patients were male. Paraprotein IgG kappa was detected in 45%, with high-risk cytogenetics [36] in 50% and a median BM infiltration of 80%. All patients had active MM disease: the sample was obtained at initial diagnosis in eight out of twelve cases, and the remaining samples were obtained at disease progression.

We observed distinct inter-individual differences in CD38 expression. In this small heterogeneous cohort, no clear association was apparent with clinical parameters, timing of sample collection (initial diagnosis vs. progressive disease under anti-myeloma therapy), or BM infiltration (Table 1).

In six out of twelve patient samples, CD38 expression increased during propagation in the 3D matrix (expression on day 9 compared to day 1, patient no. 7–12 in Figure 5A). In most samples, the addition of ATRA had no significant influence on CD38 expression (e.g., patient no. 4, Figure 5A (dotted box) and Figure 5B (left)). A significant increase after ATRA was only observed in one patient sample with already significantly higher CD38 expression due to the culture conditions alone (untreated approx. 5-fold increase compared to the first culture day vs. 8-fold after ATRA at day 9, patient no. 12, Figure 5A (dashed box) and Figure 5B (right)).

Similar findings were observed after treatment with the EZH2 inhibitor tazemetostat, with a moderate increase in CD38 in three of seven patient samples (patient no. 5, 6, and 8; Figure S5). No correlation was observed with the basal CD38 expression of the untreated cells.

Due to the small sample size and heterogeneity of this cohort—both in terms of clinical parameters and CD38 expression—the effect of daratumumab on these samples was not investigated. To address this question, a standardized collection of primary cells should be implemented as part of a future prospective study in order to compare samples from patients with an adequate response to daratumumab with those from patients who did not respond to treatment.

## 4. Discussion

Monoclonal antibodies directed against the MM surface marker CD38, such as daratumumab, are important therapeutic options for the treatment of both newly diagnosed and relapsed MM and are approved in different combinations [7,8]. However, patients may develop drug resistance despite an initial response. To restore clinical efficacy, combination with other agents is therefore relevant, and the efficacy of anti-CD38 antibodies may depend on CD38 expression on plasma cells. Various substances have demonstrated their capacity to increase CD38 expression on plasma cells, such as the HDACi panobinostat [19], the immunomodulatory drug lenalidomide [22], and the JAK2 inhibitor ruxolitinib [23]. In vitro, the enhancement of daratumumab-mediated cytotoxicity via CD38 upregulation in MM cells with epigenetic modifiers such as ATRA seemed promising [16]. This was shown both in conventional cell culture and in a humanized mouse model [16]. However, the transfer to a clinical application of this approach was less convincing [24]. To identify possible reasons for this discrepancy between conventional cell culture and the clinical setting, and to analyze the effect of different substances and their combination with daratumumab under conditions closer to the human physiological BM niche, we examined their impact on CD38 expression and cell viability in different MM cell lines under various culture conditions, and analyzed CD38 expression in primary MM patient samples after culturing in a 3D co-culture model [29]. Indeed, CD38 expression, cell viability, and response to ATRA and panobinostat treatment were specific to the individual cell line and varied depending on culture conditions. Similar differences in response to treatment with tazemetostat, depending on the cell line, have been described previously [20]. The ATRA-induced CD38 upregulation and the consequent enhanced cytotoxicity of daratumumab observed in conventional 2D culture could not be confirmed in the 3D model with stromal cell support.

The cytotoxicity of substances with anti-myeloma effects generally appears to be lower in 3D culture than in 2D culture [29]. This is not due to a lower concentration of active substances in the cell pellet of the 3D cavity, as the analyzed substances were also detected in the core of each cell aggregate [29]. Thus, the modification of CD38 expression and especially the impact on cell viability seem to depend on the individual MM cell and its interaction with the protective microenvironment of the BM niche.

As MM cells strongly depend on proliferative and anti-apoptotic signals provided by their microenvironment [37,38], in vitro models without stromal support may overemphasize the anti-myeloma effects of single substances and their combinations. In fact, the cytotoxicity of daratumumab alone also differed between the 3D model and conventional culture, with significant variations among the cell lines.

Other than this possible attenuation of a substance’s cytotoxicity due to a protective effect of the BM microenvironment, it is conceivable that, under physiological conditions, CD38 expression in myeloma cells is inherently higher than in an artificial monoculture, and that the additional administration of substances intended to upregulate CD38 has no further effect because saturation has already been reached. Indeed, we observed that CD38 expression on untreated MM cells was already moderately increased by stromal cell support alone, and a possible further increase in CD38 expression due to ATRA pretreatment became less relevant for treatment efficacy. This may be explained by enhanced proliferation under these conditions, simulating the interaction of MM cells with the surrounding BM cells.

In line with the observation that CD38 expression is highly heterogeneous on MM cells [16,20], we detected large inter-individual differences in CD38 expression on plasma cells derived from patients with active MM disease, both in untreated samples and after ATRA treatment.

Various mechanisms leading to CD38 upregulation by ATRA are discussed. The CD38 gene contains a retinoic-acid-responsive element, and the retinoic acid receptor probably plays a role in ATRA-mediated CD38 induction [15]. Additionally, non-classical retinoic acid signaling seems to be involved in CD38 upregulation [39].

Different mechanisms of action have also been demonstrated for the effect of EZH2 inhibition on CD38 expression. On the one hand, it has been shown that the CD38 promoter region itself is controlled by the chromatin-modifying enzyme EZH2. In its active form, CD38 expression is suppressed; EZH2 inhibition lifts this epigenetic brake, restoring CD38 production to the cell surface [20]. On the other hand, Liu et al. state that loss of the histone demethylase KDM6A in MM cells leads to an increase in repressive H3K27me3 methylation markers at the CD38 and CD48 promoters, leading to subsequent downregulation of both molecules. Inhibiting EZH2 re-sensitized KDM6A-deficient MM cells to daratumumab-mediated cytotoxicity through H3K27me3 inhibition and upregulation of CD38 and CD48 [21].

The interaction of various combination partners and their potential impact on CD38 expression and viability of myeloma cells is complex. For example, it has been shown that lenalidomide leads to increased CD38 expression in MM cell lines via degradation of Ikaros and Aiolos, and that the additional effect of a combination with daratumumab correlates with increased CD38 expression on MM cells [22]. However, lenalidomide had no additional effect on daratumumab-mediated cytotoxicity but enhanced the anti-myeloma effect of daratumumab by stimulating NK cells without modifying CD38 expression on these cells [22]. Thus, the efficacy of anti-CD38 antibodies may not only depend on the CD38 expression of MM cells themselves, but also on the microenvironment: CD38 is likewise expressed at different levels on surrounding cells in the BM, such as NK cells and monocytes. CD38 expression has also been described in a subgroup of regulatory T cells, and the proportion of cells highly expressing CD38 appears to be higher in MM patients than in healthy individuals [40]. Moreover, CD38 was detected on progenitors of osteoclasts, but not on mature osteoclasts after in vitro differentiation [41], and it was shown that daratumumab inhibits the formation and activity of osteoclasts, indicating the involvement of CD38 in bone remodeling in MM [41]. Osteoclasts also have an immunosuppressive impact on T cells, in turn inducing osteoclastogenesis. It has been demonstrated in vitro that an anti-CD38 antibody can enhance the cytotoxic T cell effect on osteoclasts and restore T cell proliferation, suggesting that anti-CD38 treatment may affect MM-induced bone disease [42].

This pleiotropic role of CD38 in the BM niche seems to explain the individual influence of CD38 upregulation on the viability of myeloma cells and emphasizes the artificial conditions in a conventional 2D cell culture without stromal cell support.

Other than the complex interactions of MM cells with the BM niche, which challenge a monocausal relationship between CD38 upregulation and plasma cell viability, the detection method for CD38 expression should be critically analyzed. After treatment with anti-CD38 antibodies such as daratumumab, the therapeutic antibody may interfere with the diagnostic antibody used to detect plasma cells via flow cytometry, thus hampering the appropriate identification of plasma cells and the measurement of CD38 expression on these cells. To overcome this interference, we repeated our analyses with a multi-epitope anti-CD38 antibody [43]. This approach is also pursued in clinical applications and has been included in the MM MRD panel by the EuroFlow consortium [44]. Indeed, higher CD38 levels were detected after daratumumab treatment with this approach, but interestingly also after treatment with ATRA alone. The moderate CD38 upregulation by tazemetostat, previously described [20,21] and reproduced with the monoclonal antibody, could not be detected when staining with a multi-epitope antibody, neither after tazemetostat alone nor in combination with daratumumab.

Another approach to circumvent this interference in plasma cell detection would be the use of the monoclonal antibody VS38, which can recognize a nonglycated type II transmembrane protein in plasma cells [45]. With the help of this staining, it could be shown that suppressed CD38 expression on plasma and NK cells after daratumumab treatment was more likely caused by this interference than by real antigen loss [46].

Despite the discussed advantages of the 3D co-culture system in terms of the transferability of in vitro data to the clinical setting, the model does, however, have clear limitations.

First, the experimental setup uses fixed effector-to-target ratios, but the chosen ratio does not necessarily reflect the real conditions in the BM niche. NK and MM cells were cultured together; however, uniform immune cell infiltration is not guaranteed under these conditions and was not verified in the current approach, nor was the possibility of effector cell exhaustion during long-term culture.

Second, continuous exposure to a drug at a fixed dose in vitro may either over- or underestimate therapeutic efficacy compared to the transient exposure experienced in vivo due to dynamic absorption, distribution, metabolism, and excretion profiles. Furthermore, varying therapeutic concentrations achieved in the BM, depending on the degree of MM infiltration and general perfusion, and the repeated administration as part of the treatment protocols established in the clinical trials, based, among other factors, on toxicity, cannot be simulated in this model.

Third, a valid positive control is lacking. The results could be substantially strengthened if there were a combination therapy, consisting of an anti-CD38 antibody and a CD38-upregulating substance, that is effective in clinical practice and that would then also have to be proven effective in the 3D model.

## 5. Conclusions

In MM, the modification of CD38 expression on plasma cells by epigenetic modifiers and the possible enhancement of the cytotoxic effect of anti-CD38 antibodies, such as daratumumab, seem to depend on the individual plasma cell and its interaction with the BM microenvironment. Our data are consistent with the lack of clinical efficacy of a combination treatment with daratumumab, although it was shown, for ATRA, to increase CD38 expression and cytotoxicity in a conventional 2D cell culture of MM cells. Thus, the 3D co-culture system with stromal cell support seems to be a valuable and more realistic model of the BM niche and may better predict the expected effects of various anti-myeloma substances and their combinations in vivo. This should be evaluated for correlations with clinical response as part of a future prospective sample collection.

## Figures and Tables

**Figure 1:**
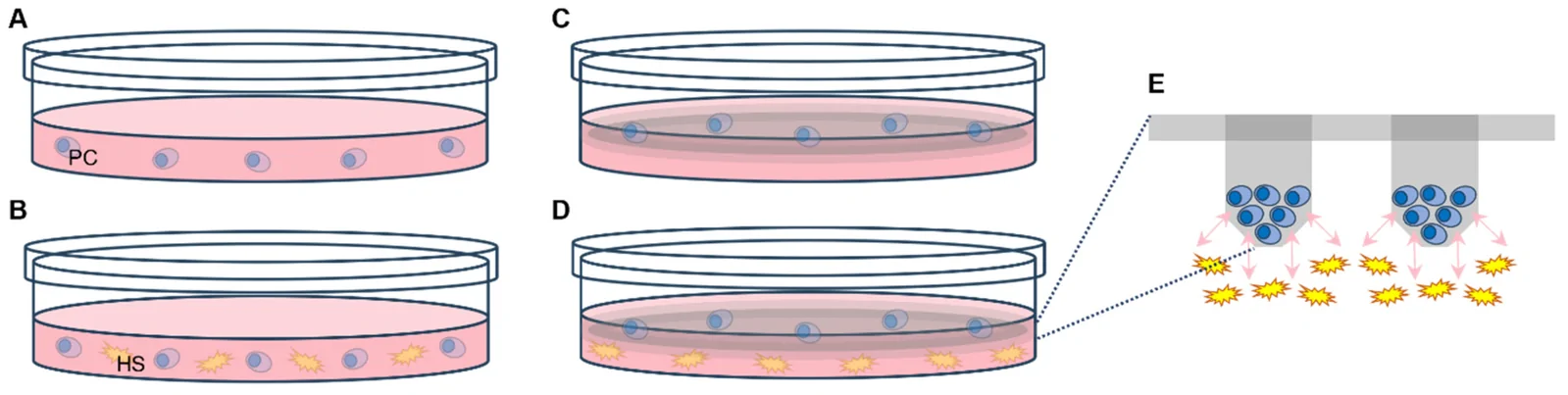
Schematic overview of culture conditions. For 2D culture, the multiple myeloma cell lines MM-1S, OPM-2, and L363 were cultured either separately in conventional cell culture (**A**) or in co-culture with the human stromal cell lines HS-5 and HS-27 in a shared dish (**B**). For 3D culture, myeloma cells were seeded into the conical microwells of an agarose-based membrane (CoSeedis™, **C**). For distance co-culture, this microwell array was placed onto confluent stromal cells (**D**). Membrane permeability allows for diffusion of cytokines between plasma and stromal cells, as shown schematically in the enlarged view (**E**). Abbreviations: PC = plasma cell; HS = bone marrow stromal cell.

**Figure 2:**
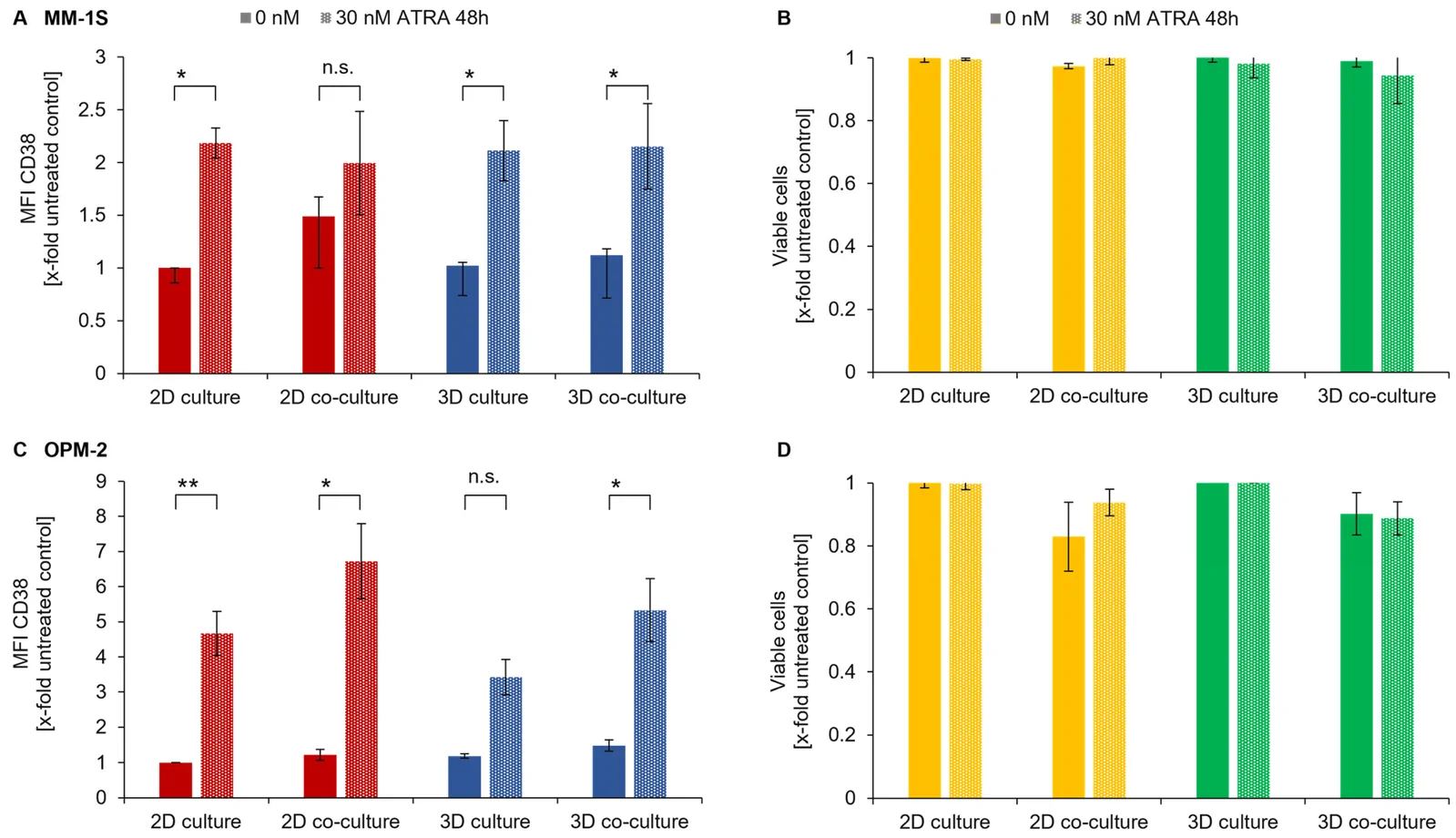
Impact of ATRA on CD38 expression and viability of MM cell lines in 2D vs. 3D culture models. MM-1S (**A**,**B**) and OPM-2 (**C**,**D**) were cultured in conventional cell culture alone or with stromal cell support (HS-5), and in a conical agarose 3D culture alone or with HS-5, and treated with ATRA. CD38 expression (**A**,**C**) and cell viability by DAPI staining (**B**,**D**) were analyzed using flow cytometry 48 h after exposure. MM-1S: n = 5; OPM-2: n = 3, except n = 2 for 3D culture alone. Abbreviations: MFI = mean fluorescence intensity; ATRA = all-*trans* retinoic acid; * *p* < 0.05; ** *p* < 0.01; n.s. = not significant.

**Figure 3:**
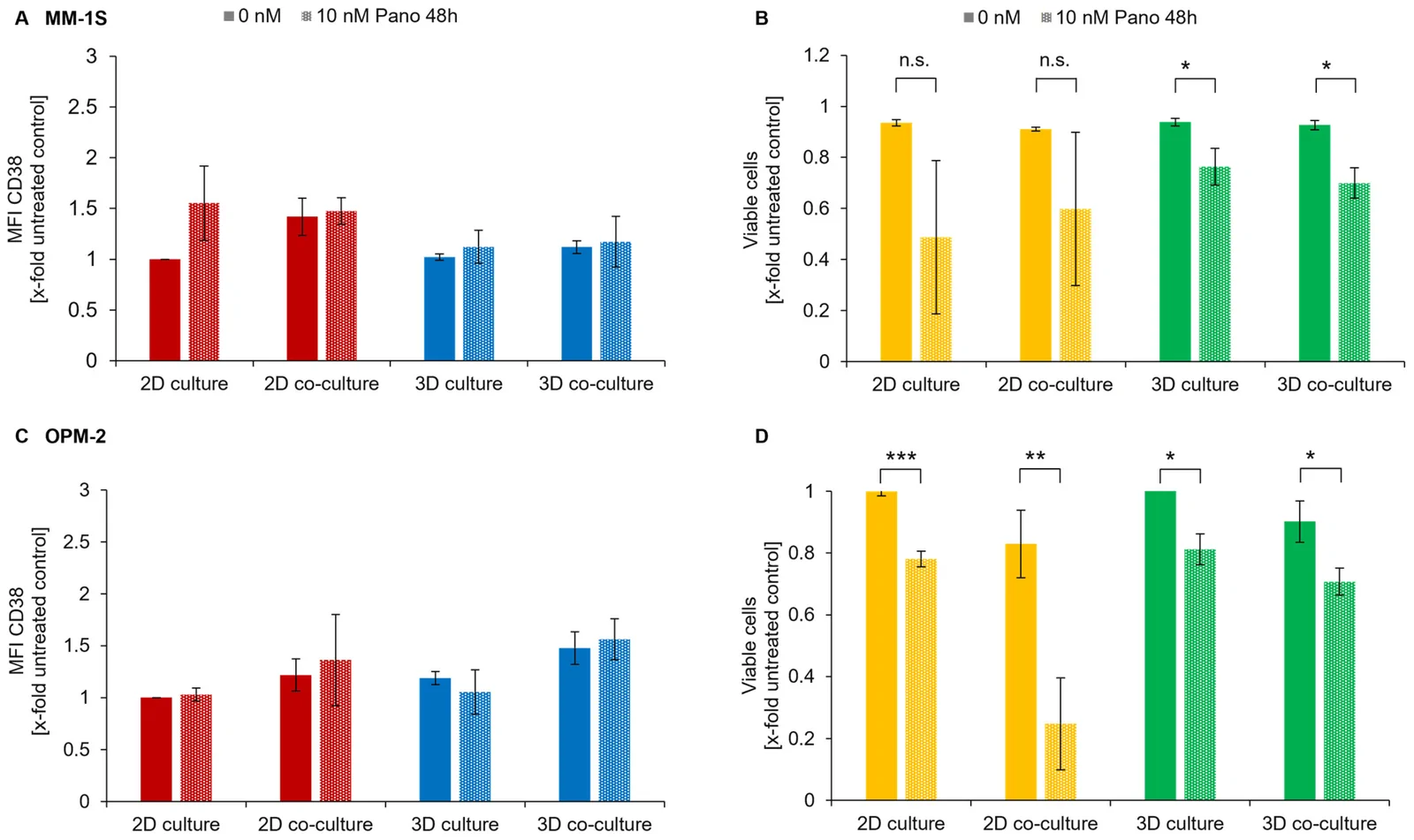
Impact of panobinostat on CD38 expression and viability of MM cell lines in 2D vs. 3D culture models. MM-1S (**A**,**B**) and OPM-2 (**C**,**D**) were cultured in conventional cell culture alone or with stromal cell support (HS-5), and in a conical agarose 3D culture alone or with HS-5, and treated with panobinostat. CD38 expression (**A**,**C**) and cell viability by DAPI staining (**B**,**D**) were analyzed using flow cytometry 48 h after exposure. MM-1S: n = 3 for 3D (co-)culture, n = 5 for 2D (co-)culture; OPM-2: n = 3, except n = 2 for 3D culture alone. Abbreviations: MFI = mean fluorescence intensity; pano = panobinostat; * *p* < 0.05; ** *p* < 0.01; *** *p* < 0.001; n.s. = not significant.

**Figure 4:**
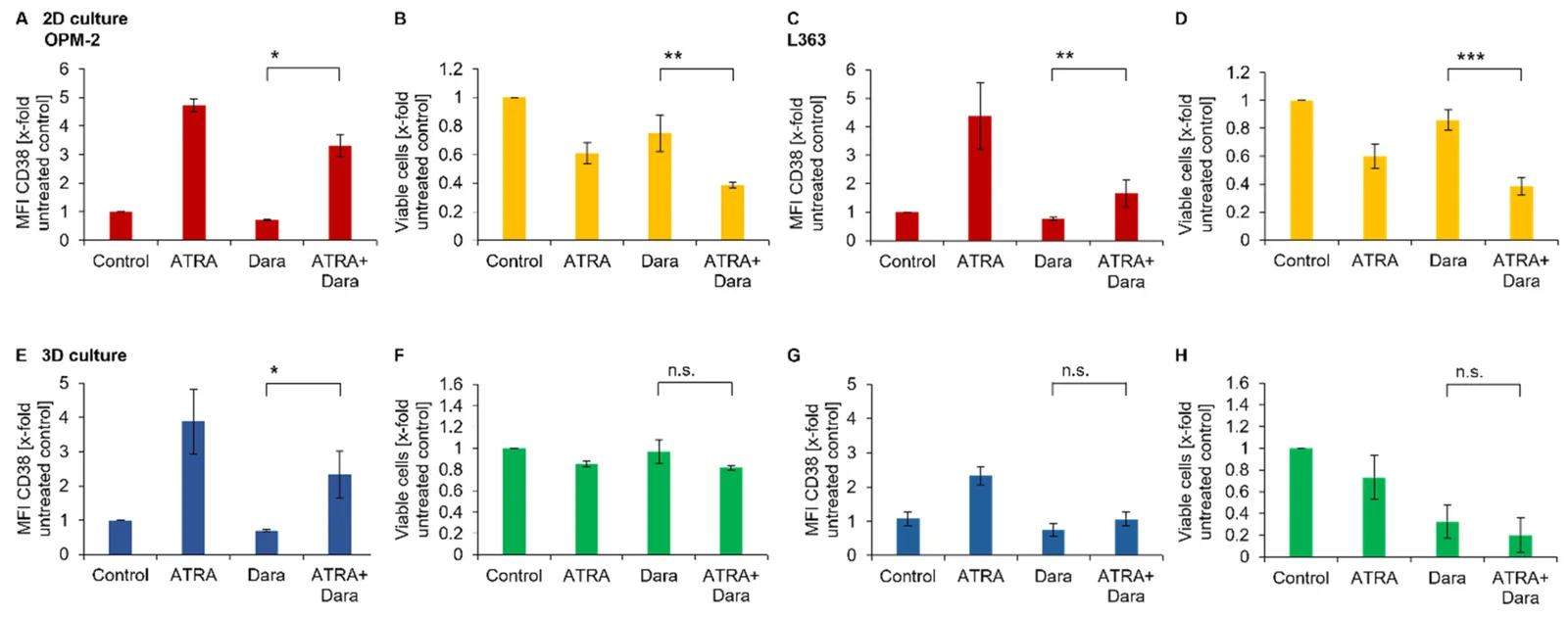
Combination treatment with daratumumab and ATRA in MM cell lines in 2D vs. 3D culture models. OPM-2 (**A**,**B**,**E**,**F**) and L363 (**C**,**D**,**G**,**H**) were cultured in conventional cell culture (**A**–**D**) and in conical agarose 3D culture (**E**–**H**) with stromal cell support (HS-5) and NK cells, and treated with daratumumab and ATRA. CD38 expression was measured with a multi-epitope anti-CD38 antibody (**A**,**E**,**C**,**G**); cell viability was measured by DAPI staining (**B**,**F**,**D**,**H**) and flow cytometry (72 (ATRA) resp.) 12 h (daratumumab) after exposure. n = 3 for OPM-2; n = 2 for L363. Abbreviations: MFI = mean fluorescence intensity; ATRA = all-*trans* retinoic acid; dara = daratumumab; * *p* < 0.05; ** *p* < 0.01; *** *p* < 0.001; n.s. = not significant.

**Figure 5:**
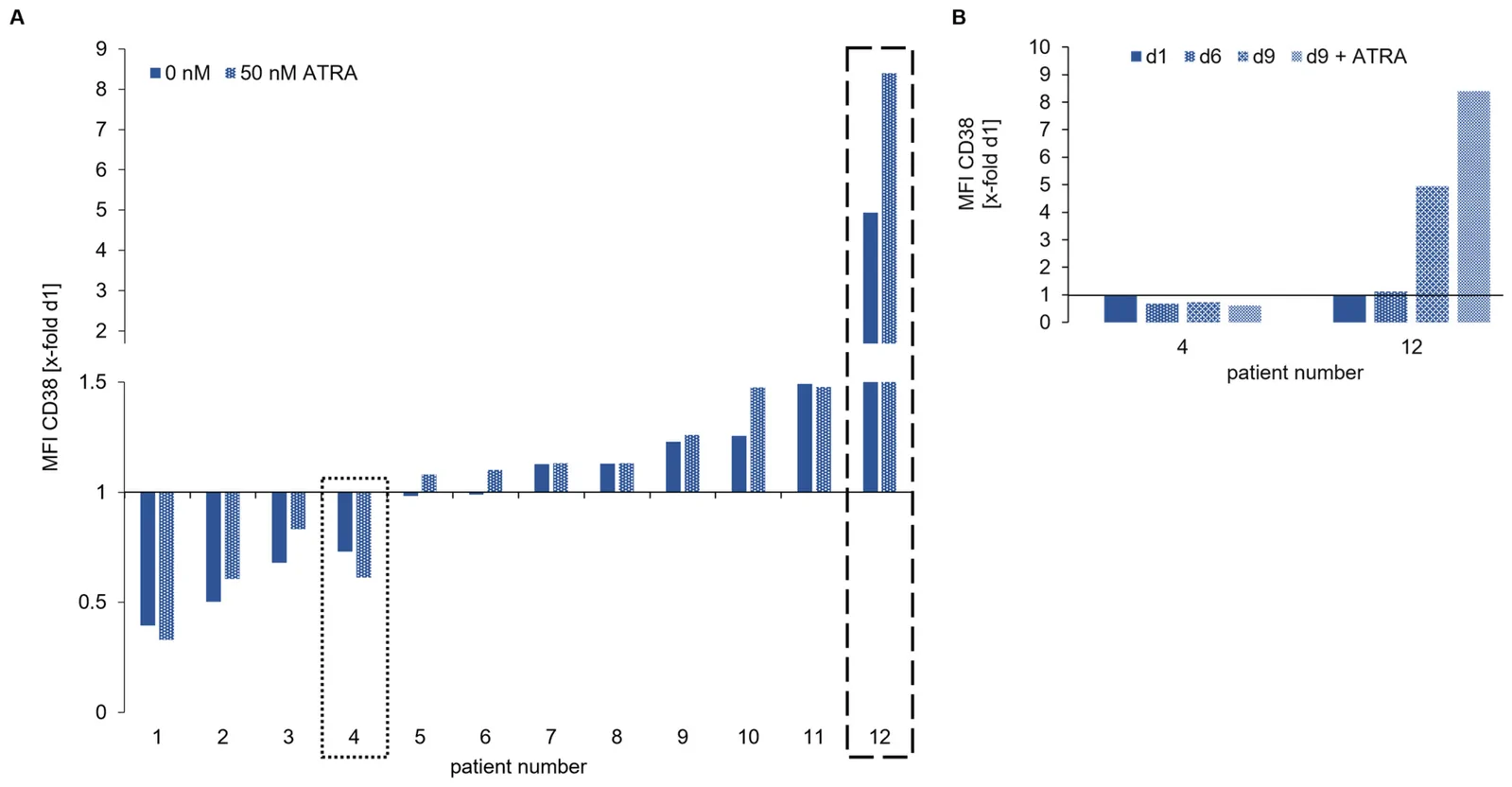
Impact of ATRA on CD38 expression in primary MM cells in 3D co-culture. (**A**) Primary patient samples were cultured in a conical agarose 3D culture with stromal cell support (HS-5) for nine days and treated with ATRA. CD38 expression was analyzed by flow cytometry 48 h after exposure. (**B**) Time-dependent course of CD38 expression, exemplified by a patient with a decrease in CD38 expression (dotted box in (**A**)) and one with a relevant increase in CD38 expression (dashed box in (**A**)) under culture conditions and after the addition of ATRA. Abbreviations: MFI = mean fluorescence intensity; ATRA = all-*trans* retinoic acid.

**Table 1:** Patient characteristics.

	Age [Ys]	Sex	MM Paraprotein	MM Stage (ISS)	CG	Remission State	Used Material	BM Infiltration [%]		IHC		MFI CD38
								IHC	Cytological	CD38	CD138	
**1**	67	m	kappa LC	I	SR	PD **	BM	30–40	40–50	pos	pos	4510
**2**	63	m	IgG kappa	III	n.e.	ID	BM	65	70	pos	pos	2210
**3**	66	m	IgG kappa	III	HR	PD * (PCL)	PE	90	n.e.	pos	pos	1664
**4**	71	m	IgG kappa	III	SR	ID	BM	60	80	pos	pos	2214
**5**	71	f	IgA kappa	I	HR	ID	BM	30	80	pos	n.e.	2741
**6**	84	m	IgG kappa	III	HR	ID	BM	30	80	pos	n.e.	662
**7**	56	m	IgM kappa	PCL	n.e.	ID	BM	80	80	pos	n.e.	1581
**8**	65	m	IgA kappa	II	HR	ID	BM	90	n.e.	pos	n.e.	447
**9**	41	f	kappa LC	III	HR	ID	BM	70	87	pos	pos	571
**10**	62	m	IgG kappa	I	SR	PD **	BM	30–40	30–40	pos	n.e.	1913
**11**	59	f	IgG lambda	III	SR	PD **	BM	20–30	20	pos	pos	3713
**12**	54	m	IgA kappa	III	SR	ID	BM	80	80	pos	pos	762

* daratumumab pretreatment, after autologous and allogeneic stem cell transplantation. ** no daratumumab pretreatment, after autologous stem cell transplantation. Cytogenetic information was not available for two samples because the available BM material was insufficient for that purpose. In two cases, BM infiltration could not be assessed cytologically due to dry tap BM aspiration (punctio sicca), presumably due to very high plasma cell infiltration, as demonstrated by IHC. In five cases, no IHC analysis of CD138 expression was available because this test is not routinely performed according to the laboratory’s internal standards at our center. Abbreviations: Ys = years; m = male; f = female; Ig = immunoglobulin; LC = light chain; ISS = International Staging System; CG = cytogenetics; n.e. = not evaluated; SR = standard risk; HR = high risk according to the International Myeloma Society (IMS) and International Myeloma Working Group (IMWG) Consensus Genomic Staging (CGS) [36]; PD = progressive disease; ID = initial diagnosis; PCL = plasma cell leukemia; BM = bone marrow; PE = pleural effusion; IHC = immunohistochemistry, MFI = mean fluorescence intensity, pos = positive.

## Data Availability

The original contributions presented in this study are included in the article and Supplementary Materials. Further inquiries, such as flow cytometry gating plots, can be directed to the corresponding author.

## Supplementary Materials

The following supporting information can be downloaded at: https://www.mdpi.com/article/10.3390/cancers18193093/s1, Figure S1: CD38 upregulation remains cell line-dependent also after long-term cultivation with a corresponding influence on cell viability; Figure S2: The impact of stromal support on CD38 upregulation is largely independent from the stroma cell line or concentration of supernatant obtained from stroma cell culture; Figure S3: Comparison of monoclonal versus multi-epitope anti-CD38 antibody; Figure S4: EZH2 inhibition with tazemetostat has no relevant influence on CD38 expression or cell viability when combined with daratumumab in 3D co-culture; Figure S5: Impact of tazemetostat on CD38 expression in primary MM cells in 3D co-culture.

## Abbreviations

The following abbreviations are used in this manuscript:

ADCC	Antibody-dependent cellular cytotoxicity
ATRA	All-*trans* retinoic acid
BM	Bone marrow
CAR	Chimeric antigen receptor
CDC	Complement-dependent cytotoxicity
CG	Cytogenetics
CGS	Consensus Genomic Staging
dara	daratumumab
EZH2	enhancer of zeste homolog 2
f	female
HDACi	pan–histone deacetylase inhibitor
HS	Bone marrow stromal cell
ID	Initial diagnosis
Ig	Immunoglobulin
IMS	International Myeloma Society
IMWG	International Myeloma Working Group
ISS	International Staging System
LC	Light chain
MFI	Mean fluorescence intensity
m	male
MM	Multiple Myeloma
n.e.	not evaluated
NK	natural killer
n.s.	not significant
ORR	Overall response rate
pano	panobinostat
PB	Peripheral blood
PC	Plasma cell
PCL	Plasma cell leukemia
PD	Progressive disease
PE	Pleural effusion
pos	positive
SR	Standard risk
HR	High risk
Ys	Years
