# Supplementary material for: An Ex Vivo 3D Co-Culture Model of the Bone Marrow Niche Better Predicts Clinically Relevant Epigenetic Target Modulation to Improve Immunotherapy in Multiple Myeloma
Source: Cancers (Basel). 2026 Sep 23;18(19):3093. doi: 10.3390/cancers18193093 (PMC13664379; doi:10.3390/cancers18193093)
Supplement: Supplementary file 1 [file cancers-18-03093-s001.zip › cancers-4542605-supplementary.pdf]

Supplementary Material

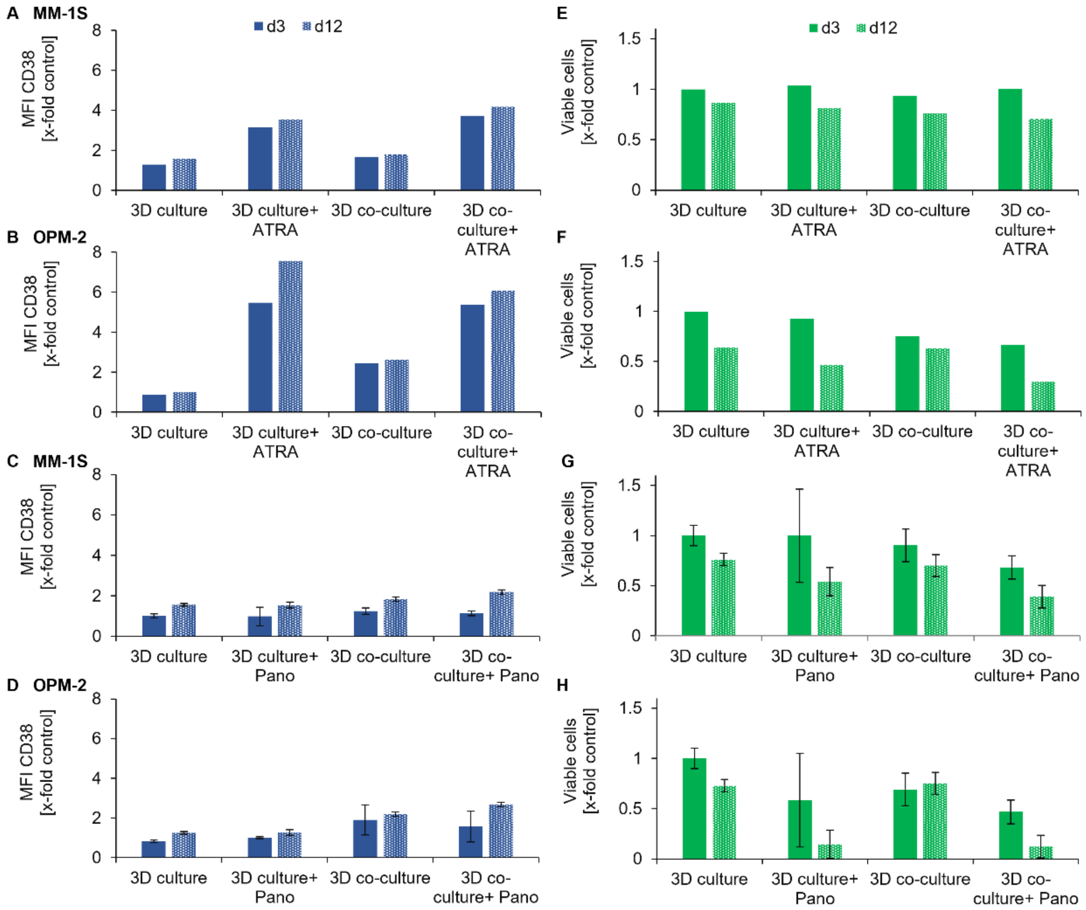

Figure S1. CD38 upregulation remains cell line-dependent also after long-term cultivation with a corresponding influence on cell viability.

MM-1S (**A, C** and **E, G**) and OPM-2 (**B, D** and **F, H**) were cultured in a conical agarose 3D culture alone or with stromal cell support (HS-5) and treated with ATRA (**A, B** and **E, F**) and panobinostat (**C, D** and **G, H**). CD38 expression (**A - D**) and cell viability by DAPI-stain (**E - H**) were analyzed by flow cytometry 3 and 12 days after exposure. n = 2 for pano, n =1 for ATRA, control = untreated 2D culture without stromal cell support. *Abbreviations*: MFI = mean fluorescence intensity; ATRA = all-*trans* retinoic acid; pano = panobinostat.

In contrast to the only transient increase of CD38 expression on peripheral blood immune cell subsets described by Frerichs et al. [24], ATRA and less pronounced panobinostat led to a continuous increase of CD38 expression from day 3 to day 12 in the 3D model with or without stromal support in both MM-1S and OPM-2. In addition to the obtained CD38 upregulation, there was a more pronounced long-term effect on viability than after a short culture period, with reduced cell viability as compared to the untreated long-term culture after treatment with ATRA or panobinostat on day 12.

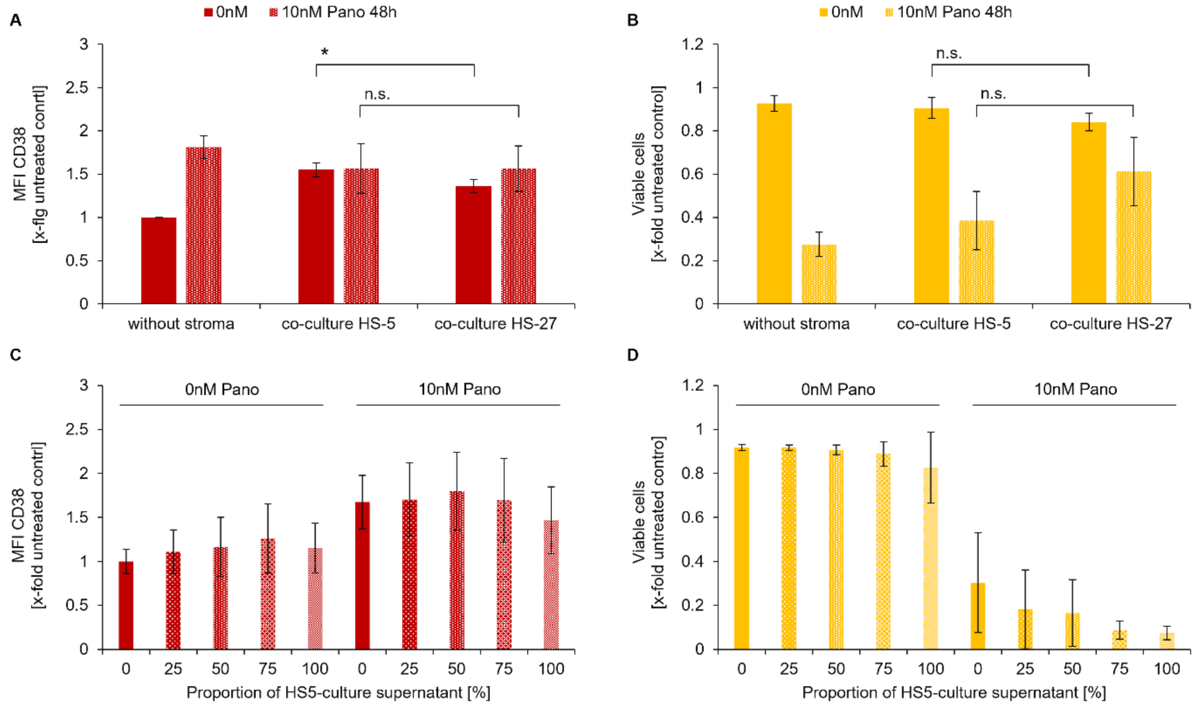

Figure S2. The impact of stromal support on CD38 upregulation is largely independent from the stroma cell line or concentration of supernatant obtained from stroma cell culture.

MM-1S were cultured in conventional cell culture, in co-culture with HS-5 and HS-27 (**A, B**), respectively, or with concentration series of supernatant obtained from HS-5 culture (**C, D**) and treated with panobinostat as indicated. CD38 expression (**A, C**) and cell viability by DAPI-stain (**B, D**) were analyzed by flow cytometry 48 hours after exposure. n=3. *Abbreviations*: MFI = mean fluorescence intensity; pano = panobinostat; * $p < 0.05$; n.s. = not significant.

Due to known differences between various BM-derived cell lines [20], we compared co-culture of MM-1S with HS-5 versus HS-27 without treatment and after panobinostat with its known effect on cell viability. We observed a slightly higher CD38-expression of untreated MM-1S cells in co-culture with HS-5 as compared to HS-27 (1.55- versus 1.36-fold increase in relation to the control without stromal support; **A**, filled bars) and a stronger cytotoxic effect of panobinostat in co-culture with HS5 (reduction to 39% versus 61% of untreated control with HS-27; **B**, patterned bars). These observations are consistent with the fact that HS-27 are lesser secreting growth factors and thus may not support the proliferation of primary MM cells in serum-deprived media without exogenous factors [31]. We therefore chose HS-5 for further experiments.

To investigate the influence of stromal cell cytokines independently of any possible influence on growth due to interaction between stroma and myeloma cells under culture conditions, MM-1S were treated in conventional cell culture with addition of supernatant obtained from HS-5 culture. The concentration of the applied supernatant had no impact on CD38 expression, neither in untreated cells (**C** left) nor after addition of panobinostat (**C** right). In untreated cells, the concentration of the supernatant likewise had no effect on cell viability (**D** left). After panobinostat treatment, there was a trend toward reduced viability with increasing supernatant concentration (reduction to 18% of untreated control with medium with 25% supernatant versus 9% with 75% supernatant; **D** right) possibly indicating an increase in efficacy under cytokine influence. However, the differences between the various concentration levels were not statistically significant. In summary, the secreted cytokines of stroma cells alone without direct crosstalk between myeloma and stroma cells had no impact on CD38 expression.

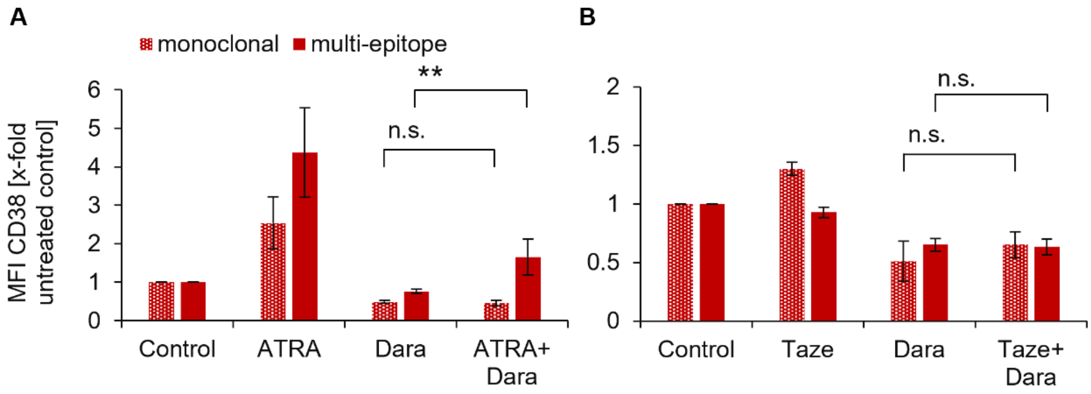

Figure S3. Comparison of monoclonal versus multi-epitope anti-CD38 antibody.

L363 were cultured in conventional cell culture with stromal cell support (HS-5) and NK cells and treated with daratumumab and ATRA (**A**) or tazemetostat (**B**). CD38 expression was measured both with a monoclonal antibody (patterned bars) and with a multi-epitope anti-CD38 antibody. n = 2. *Abbreviations*: MFI = mean fluorescence intensity; ATRA = all-*trans* retinoic acid; taze = tazemetostat; dara = daratumumab; ** $p < 0.01$; n.s. = not significant.

In conventional 2D co-culture of L363 with NK cells and stromal support, higher CD38 expression was detected on plasma cells pretreated with daratumumab when using a multi-epitope as compared to a conventional monoclonal anti-CD38 antibody (49% versus 76% of untreated control; **A**, patterned versus filled bars). Measurements with the multi-epitope antibody also revealed higher CD38 values after treatment with ATRA alone and in combination with daratumumab as compared to staining with the conventional monoclonal antibody.

In line with published data [20,21], in case of flow cytometric measurement using a monoclonal anti-CD38 antibody, treatment with tazemetostat in conventional co-culture with NK cells and stromal support cells led to a moderate increase in CD38 expression (1.3-fold; **B**, patterned bars). This CD38 upregulation after the addition of tazemetostat was not observed when using the multi-epitope anti-CD38 antibody (**B**, filled bars).

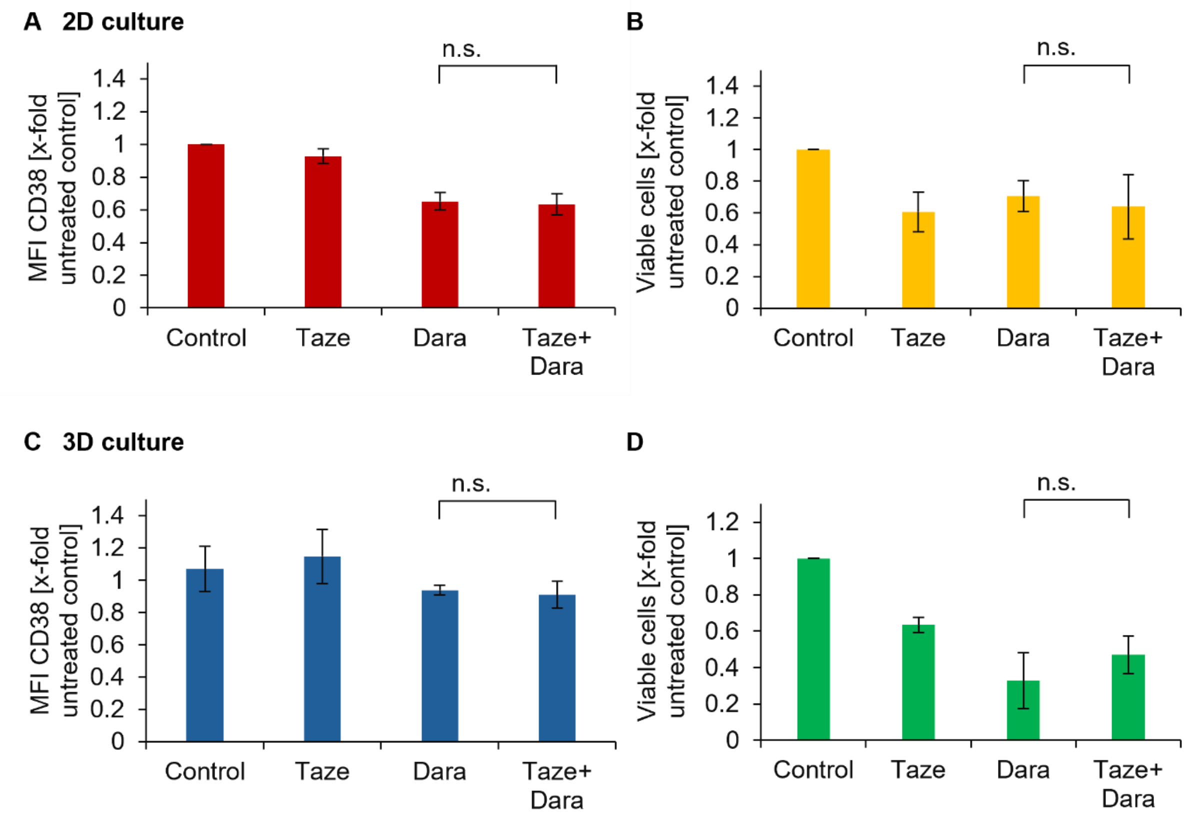

Figure S4. EZH2 inhibition with tazemetostat has no relevant influence on CD38 expression or cell viability when combined with daratumumab in 3D co-culture. L363 were cultured in conventional cell culture (**A, B**) and in conical agarose 3D culture (**C, D**) with stromal cell support (HS-5) and NK cells and treated with daratumumab and tazemetostat. CD38 expression was measured with a multi-epitope anti-CD38 antibody (**A, C**). Cell viability by DAPI-stain (**B, D**) was analyzed by flow cytometry 72 resp. 12 h after exposure. n = 2. *Abbreviations*: MFI = mean fluorescence intensity; taze = tazemetostat; dara = daratumumab; n.s. = not significant.

As it was shown in conventional cell culture that EZH2 inhibition leads to CD38 upregulation but does not improve ADCC by anti-CD38 antibodies in plasma cells with high basal CD38 expression [20], L363 with relatively low basal CD38 expression were treated with the EZH2 inhibitor tazemetostat alone and in combination with daratumumab. The combination of tazemetostat with daratumumab had neither a relevant impact on CD38 expression nor on cell viability (**A, B**). Similar results were obtained in 3D co-culture (**C, D**).

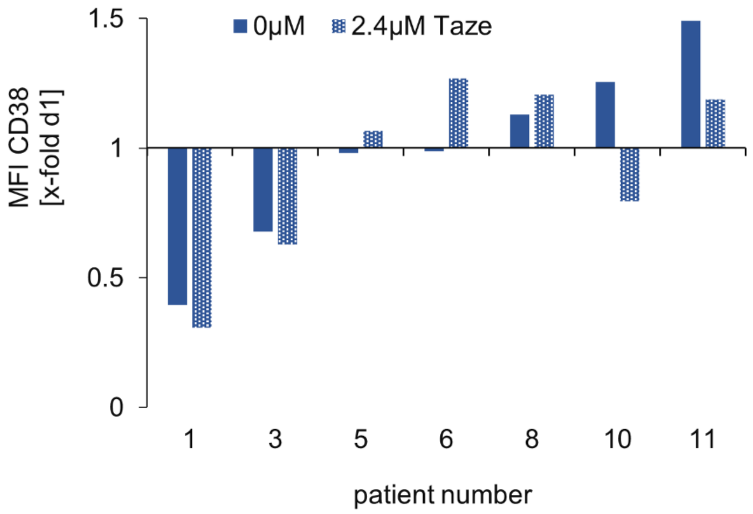

Figure S5. Impact of tazemetostat on CD38 expression in primary MM cells in 3D co-culture.

Primary patient samples were cultured in a conical agarose 3D culture with stromal cell support (HS-5) for nine days and treated with tazemetostat. CD38 expression was analyzed by flow cytometry 48 hours after exposure. *Abbreviations*: MFI = mean fluorescence intensity; taze = tazemetostat.

After treatment with the EZH2 inhibitor tazemetostat, CD38 increased at most moderately in 3 of 7 patient samples (patient no. 5, 6 and 8). No correlation was observed with the basal CD38 expression of the untreated cells.

References

Please refer to the directory in the main manuscript
